# Supplementary figures and images for: Re-visiting the potential impact of doxycycline post-exposure prophylaxis (doxy-PEP) on the selection of doxycycline resistance in Neisseria commensals
Source: bioRxiv. 2025 Jan 9:2025.01.09.632169. Preprint. [Version 1] doi: 10.1101/2025.01.09.632169 (PMC11741392; doi:10.1101/2025.01.09.632169)

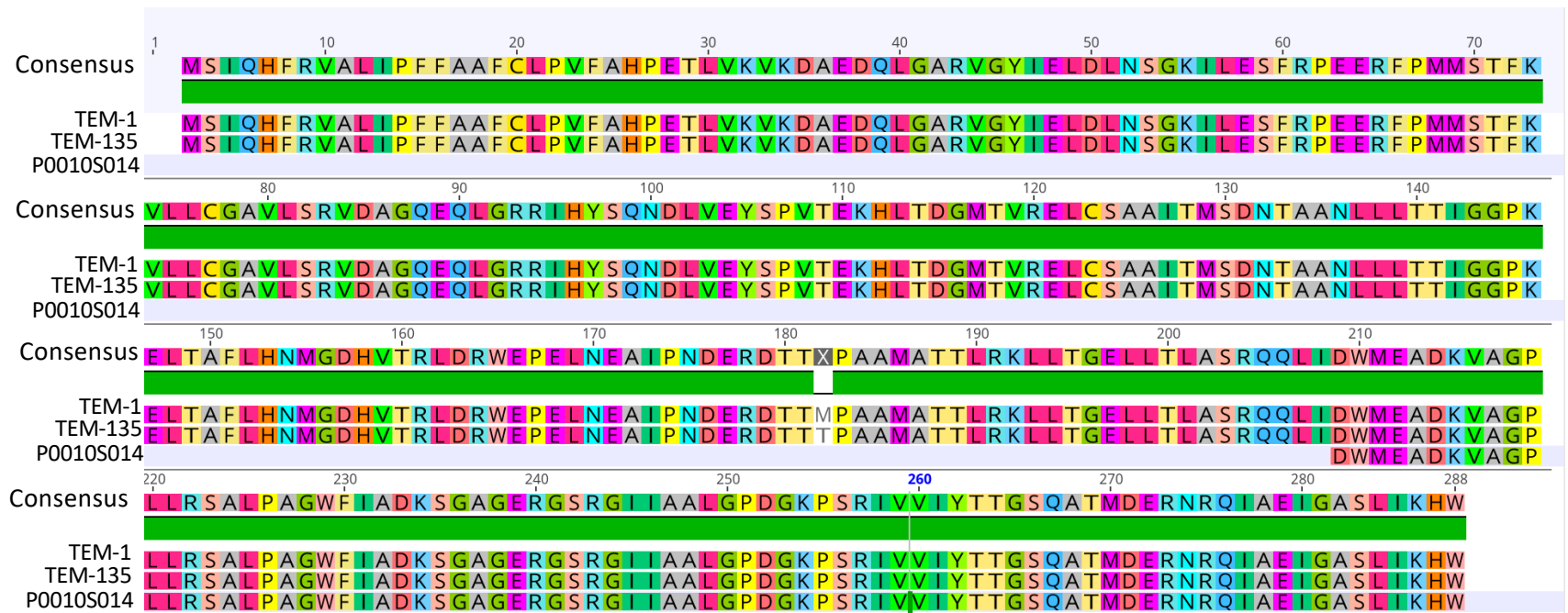

Supplement: Supplement 1 — Supplementary Figure 1. Alignment of the pbla β-lactamase gene variants TEM-1 and TEM-135. Here, we identify one novel N. subflava isolate carrying a TEM gene (Participant 10, isolate 4). The TEM gene in isolate was a partial hit to TEM-1 (allele 3) and TEM-135 (allele 2) alleles, having 100% similarity to both at nucleotide positions 619 to 891 (252 bps). This partial hit was present on a single contig in the isolate’s assembly (Contig 2584), which had a total length of 252 bps. This partial sequence did not include sites (i.e., 182) which would allow identification of the TEM allele present within this isolate. [file media-1.pdf]
